# Benefit of human moderate running boosting mood and executive function coinciding with bilateral prefrontal activation

**DOI:** 10.1038/s41598-021-01654-z

**Published:** 2021-11-22

**Authors:** Chorphaka Damrongthai, Ryuta Kuwamizu, Kazuya Suwabe, Genta Ochi, Yudai Yamazaki, Takemune Fukuie, Kazutaka Adachi, Michael A. Yassa, Worachat Churdchomjan, Hideaki Soya

**Affiliations:** 1 Laboratory of Exercise Biochemistry and Neuroendocrinology, Faculty of Health and Sport Sciences, University of Tsukuba, Ibaraki, 305-8574 Japan; 2 Sports Neuroscience Division, Department of Mind, Advanced Research Initiative for Human High Performance (ARIHHP), Laboratory of Exercise Biochemistry and Neuroendocrinology, Faculty of Health and Sport Sciences, University of Tsukuba, 1-1-1 Tennoudai, Tsukuba, Ibaraki 305-8574 Japan; 3 Faculty of Physical Therapy and Sports Medicine, Rangsit University, Pathum Thani, 12000 Thailand; 4 Faculty of Health and Sport Sciences, Ryutsu Keizai University, Ryugasaki, 301-8555 Japan; 5 Department of Health and Sports, Niigata University of Health and Welfare, Niigata, 950-3198 Japan; 6 School of Nursing and Social Services, Health Sciences University of Hokkaido, Hokkaido, 061-0293 Japan; 7 Laboratory of Applied Anatomy, Faculty of Health and Sport Sciences, University of Tsukuba, Ibaraki, 305-8574 Japan; 8 Department of Neurobiology and Behavior, University of California Irvine, Irvine, CA 92679-3800 USA; 9 Center for the Neurobiology of Learning and Memory, University of California Irvine, Irvine, CA 92679-3800 USA

**Keywords:** Neuroimmunology, Microglia, Psychiatric disorders

## Abstract

Running, compared to pedaling is a whole-body locomotive movement that may confer more mental health via strongly stimulating brains, although running impacts on mental health but their underlying brain mechanisms have yet to be determined; since almost the mechanistic studies have been done with pedaling. We thus aimed at determining the acute effect of a single bout of running at moderate-intensity, the most popular condition, on mood and executive function as well as their neural substrates in the prefrontal cortex (PFC). Twenty-six healthy participants completed both a 10-min running session on a treadmill at 50%${\dot{\text{V}}\text{O}}_{{{\text{2peak}}}}$ and a resting control session in randomized order. Executive function was assessed using the Stroop interference time from the color-word matching Stroop task (CWST) and mood was assessed using the Two-Dimensional Mood Scale, before and after both sessions. Prefrontal hemodynamic changes while performing the CWST were investigated using functional near-infrared spectroscopy. Running resulted in significant enhanced arousal and pleasure level compared to control. Running also caused significant greater reduction of Stroop interference time and increase in Oxy-Hb signals in bilateral PFCs. Besides, we found a significant association among pleasure level, Stroop interference reaction time, and the left dorsolateral PFCs: important brain loci for inhibitory control and mood regulation. To our knowledge, an acute moderate-intensity running has the beneficial of inducing a positive mood and enhancing executive function coinciding with cortical activation in the prefrontal subregions involved in inhibitory control and mood regulation. These results together with previous findings with pedaling imply the specificity of moderate running benefits promoting both cognition and pleasant mood.

## Introduction

Running is not only a principal form of physical exercise, but it also has roots as a major contributing factor to the physiological and anatomical evolution of humans^1,2^. As a whole-body locomotive movement, running may greatly impact on mental health via stimulating the brains that differ from other forms of exercise such as leg-based pedaling. Given exercise is medicine, the effects of drugs differ depending on the type of drug, different types of exercise such as running and pedaling should be observed to have different effects on mental health and brain activation as well.

Consider the previous studies, including our own, physical exercise has been reported to enhance executive function by predominantly activating the left dorsolateral prefrontal cortex (l-DLPFC) that is the brain loci implicating in inhibitory control, without reporting change of pleasant mood^3–5^. Almost these studies have used pedaling as a physical exercise, not running. Although running was reported to improve mood as well as neurocognitive function based-on event-related potentials (ERPs), but a neuronal substrate with which running enhances executive function related to prefrontal subregion activation remains unelucidated^6–8^.

Running is not only an exercise that promotes physical health by enhancing cardiovascular endurance, strengthening muscles and building strong bones as it is a weight-bearing exercise, but also has an implication in lowering mental health burden^9–12^. To execute the appropriate movement, running requires neuronal top-down feedforward control responses to multi-modal sensory information to control coordinated movements and balance^13–15^. The prefrontal cortex, a brain region implicated in cognition and mood regulation, is partially involve in running, especially when there is demand for coordinated action^14–21^. Muscles throughout the body, in particular leg muscles, are recruited continuously to propel the body forward while supporting the body weight^22^. This evidence may support that running has stronger beneficial effects on mood and executive function related to prefrontal activation compared to other forms of exercise that do not require as much coordination of weight-bearing activity such as pedaling. Furthermore, the mechanical impact of each foot-strike during running has been shown to increase blood circulation peripherally and centrally that may benefit brain activation^23,24^. However, these findings need more evidence to explain a mechanism. Besides, a mechanical force from vertical head acceleration, the rate of change of head velocity in up and down direction, during running has been shown in animal studies to induce serotonin receptor internalization in the prefrontal cortex, improving emotion and cognitive control^25^. Based on these properties of running, we hypothesized that running has the potential to enhance mood and executive function with broad prefrontal activation.

Few studies reported that a single bout running influence an inhibitory control, a core executive function which involves being able to control one’s attention, behavior, thoughts and emotions to override strong internal predispositions or external lures^16,26–28^. The color-word matching Stroop task (CWST) is extensively used in both experimental and clinical settings to measure this aspect of executive function localized in the prefrontal cortex, in particular l-DLPFC^29–31^. The CWST consisted of three conditions those are neutral, congruent and incongruent; ranging from the lowest to the highest executive function demand. The test requires the participants to name the color in which a word is written rather than reading the word itself as quickly as possible^30^. Based-on the previous evidence, we hypothesized that an acute moderate-intensity running would be associated with shorter interference times (i.e., difference between response times in neural condition vs. incongruent condition), which could reflect an enhancement of executive function.

With respect to neural mechanisms, it has remained unknow how running induced neural activation in enhancing mood and executive functions. Based-on the properties of running, running may induce arousal levels by activating the reticular-activating system (RAS), a network of neurons located in the brainstem, that regulates ascending projections to the prefrontal cortex that are implicated in cognitive control and mood regulation^32–39^. To test this assumption, we used functional near-infrared spectroscopy (fNIRS) during the performance of the CWST to measure prefrontal hemodynamic changes, as in our previous studies^3–5,40–43^.

Considering statistical robustness as regards neuroimaging analysis, we focused on comparison between resting control and running to clarify the effects of an acute bout of running with hypothesizing that acute moderate-intensity running would enhance executive function by greatly impacting on positive mood with broad prefrontal activation.

## Results

### Verification of moderate intensity running

To verify whether the participants could perform moderate-intensity running, we monitored heart rate (HR) and rate of perceived exertion (RPE) every minute^44^. During the last minute of running, average HR and RPE were 141.35 ± 11.31 bpm and 10.61 ± 1.72 points, respectively. Based on the guidelines of the American College of Sports Medicine, average HR was in the appropriate range for moderate-intensity exercise whereas RPE was observed to be lower than the reference range (12–13 points)^45^.

### Psychological results

**Figure 1:**
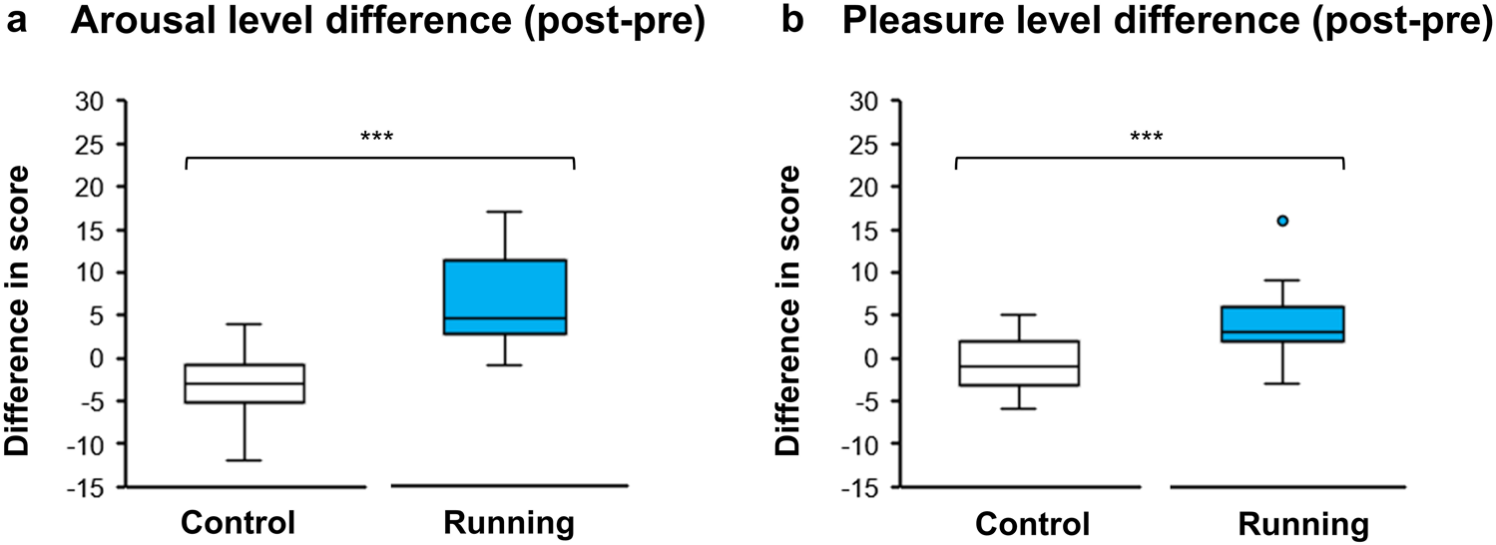
Comparison in mood change [(post-session)—(pre-session)] between control and running: (**a**) arousal level difference and (**b**) pleasure level difference. The bottom, middle and top lines of each box indicate the 25th, 50th (median) and 75th percentiles, respectively. Whiskers above and below each box refer to the most extreme point within 1.5 times the interquartile range. Points above the whiskers are outliers. *** = *p* < 0.001.

Arousal and pleasure were found to have significant interactions between session (CON, RUN) and time (pre, post) (*F*(1, 25) = 62.21, *p* < 0.001 and *F*(1, 25) = 24.73, *p* < 0.001, respectively; repeated-measures two-way ANOVA). Changes of arousal and pleasure were subsequently examined revealing that the RUN session had a significantly greater increase of arousal and pleasure than did the CON session (*t*(25) = 7.89, *p* < 0.001, Cohen’s *d* = 1.55 (Fig. 1a) and *t*(25) = 4.97, *p* < 0.001 , Cohen’s *d* = 0.98 (Fig. 1b), respectively; paired *t*-test). A comparison of baseline arousal and pleasure between CON and RUN were provided in Supplementary A.

### Behavioral results

**Figure 2:**
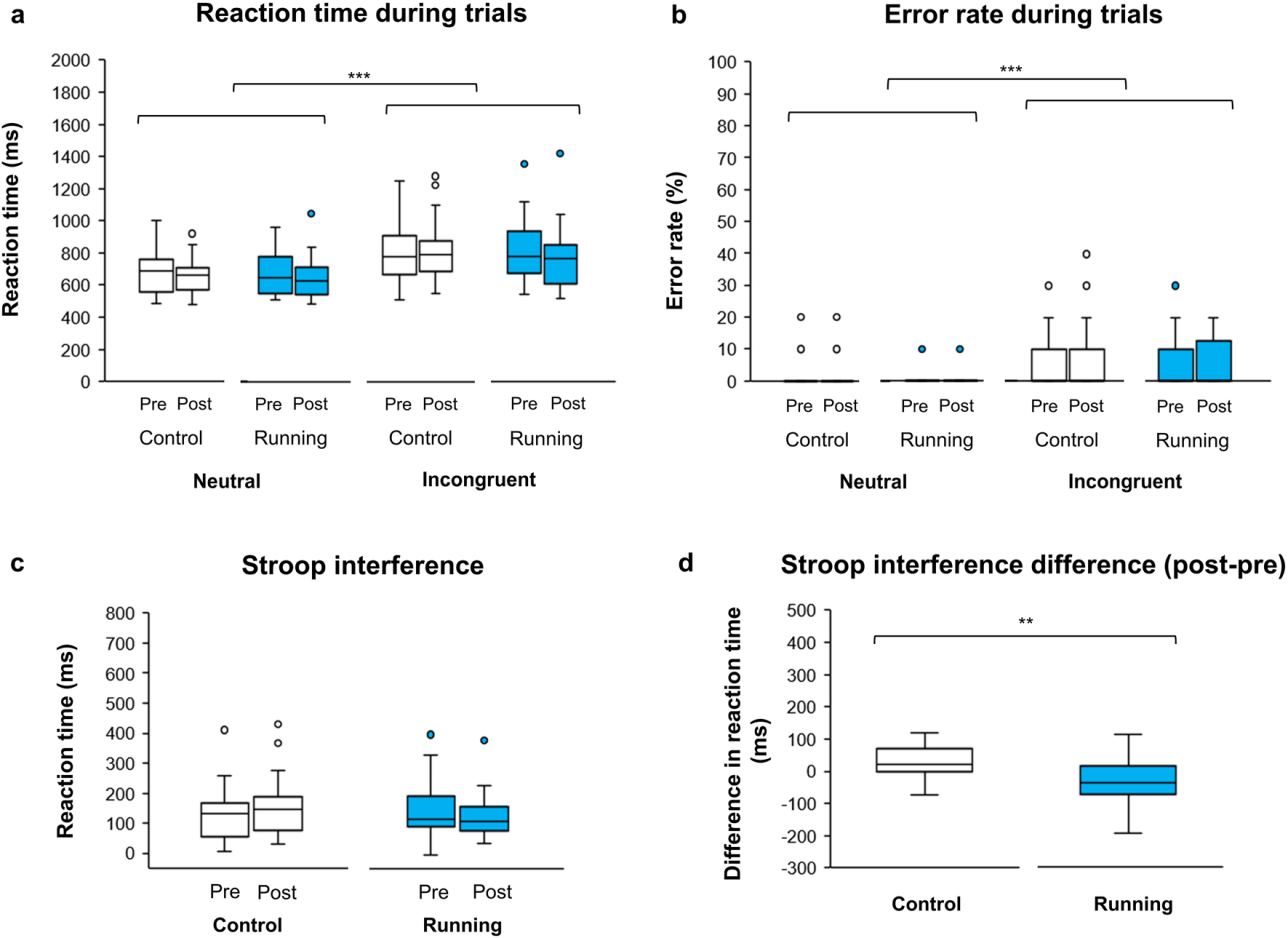
Comparison of Stroop task performance in (**a**) reaction time and (**b**) error rate between neutral and incongruent conditions. (**c**) Difference in Stroop interference [incongruent—neutral] between control and running sessions. (**d**) Contrast in Stroop interference difference ([incongruent—neutral of post-session]—[incongruent—neutral of pre-session]) between control and running. The bottom, middle and top lines of each box indicate the 25th, 50th (median) and 75th percentiles, respectively. Whiskers above and below each box refer to the most extreme point within 1.5 times the interquartile range. Points above the whiskers are outliers. *** = *p* < 0.001, ** = *p* < 0.01.

RT and ER had significant main effects of condition (neutral/incongruent; *F*(1, 25) = 87.47, *p* < 0.001 (Fig. 2a) and *F*(1, 25) = 27.50, *p* < 0.001 (Fig. 2b), respectively; repeated-measures three-way ANOVA) showing that the Stroop interference effect was basically found between the neutral and incongruent conditions in all sessions of this study. Next, the effects of running on Stroop interference RT and ER were examined. Stroop interference RT revealed significant interaction between session (CON, RUN) and time (pre, post) (*F*(1, 25) = 11.79, *p* = 0.002; repeated-measures two-way ANOVA; Fig. 2c) whereas Stroop interference ER did not reveal significant interaction (*F*(1, 25) = 0.013, *p* = 0.911). Finally, changes in Stroop interference RT were investigated and the results showed that Stroop interference RT in the RUN session was significantly more reduced than in the CON session (*t*(25) = -3.43, *p* = 0.002, Cohen’s *d* = 0.67; paired *t*-test; Fig. 2d).

### fNIRS results

**Figure 3:**
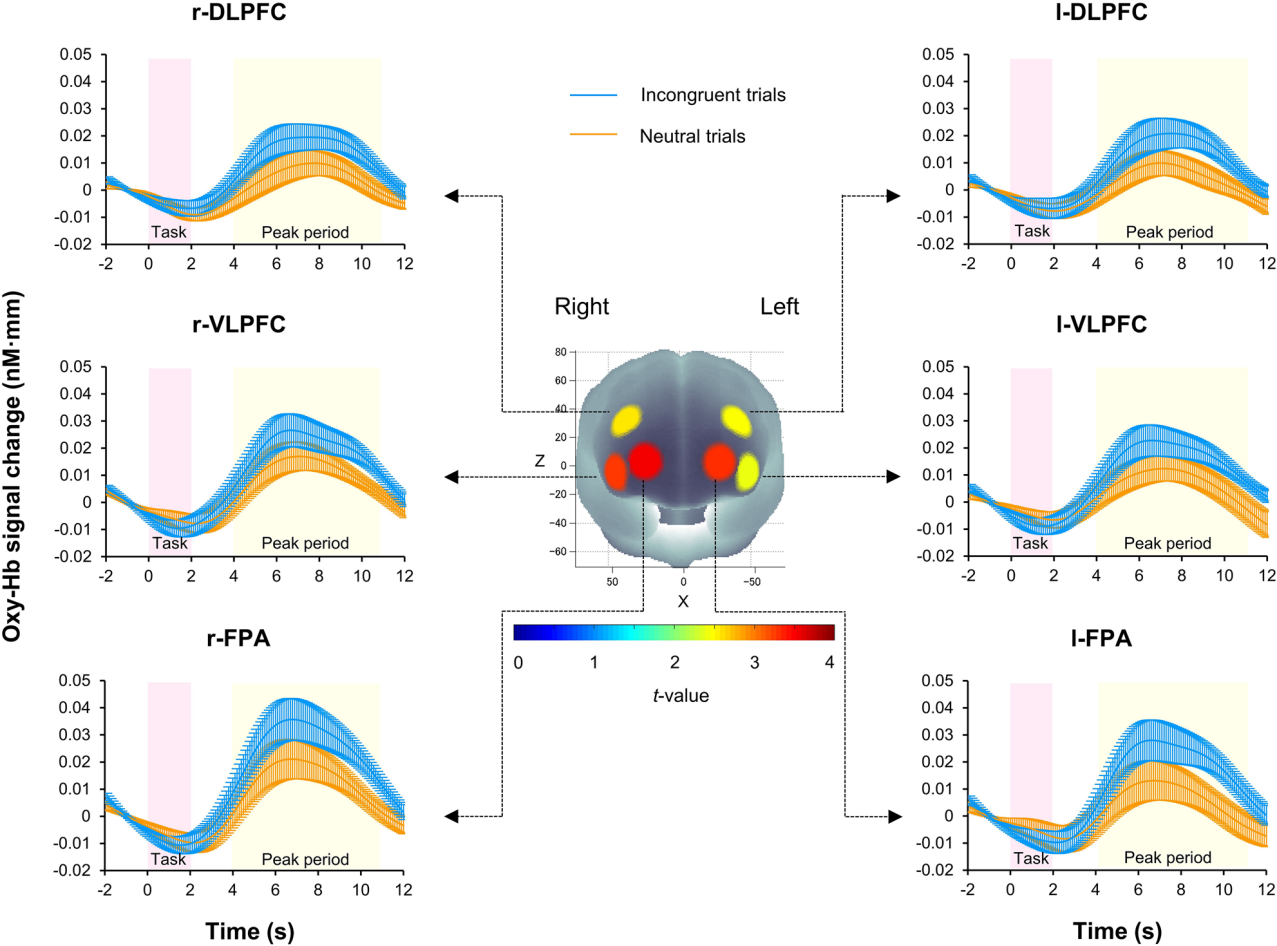
Cortical activation patterns during performance of the color-word matching Stroop task (CWST) in the pre-session of control and running. Presented data are average values between the pre-session of control and the pre-session of running. Baseline (2 s before trial onset) is set at zero and peak periods are indicated from 4 to 11 s after trial onset. Significant Stroop-interference-related cortical activations [incongruent—neutral] were found in all regions of interest (ROIs) indicating that the Stroop interference effect can be observed. The *t*-map demonstrates oxygenated hemoglobin (Oxy-Hb) signal change, *t*-values are indicated as in the color bar. Data are mean ± SE.

**Figure 4:**
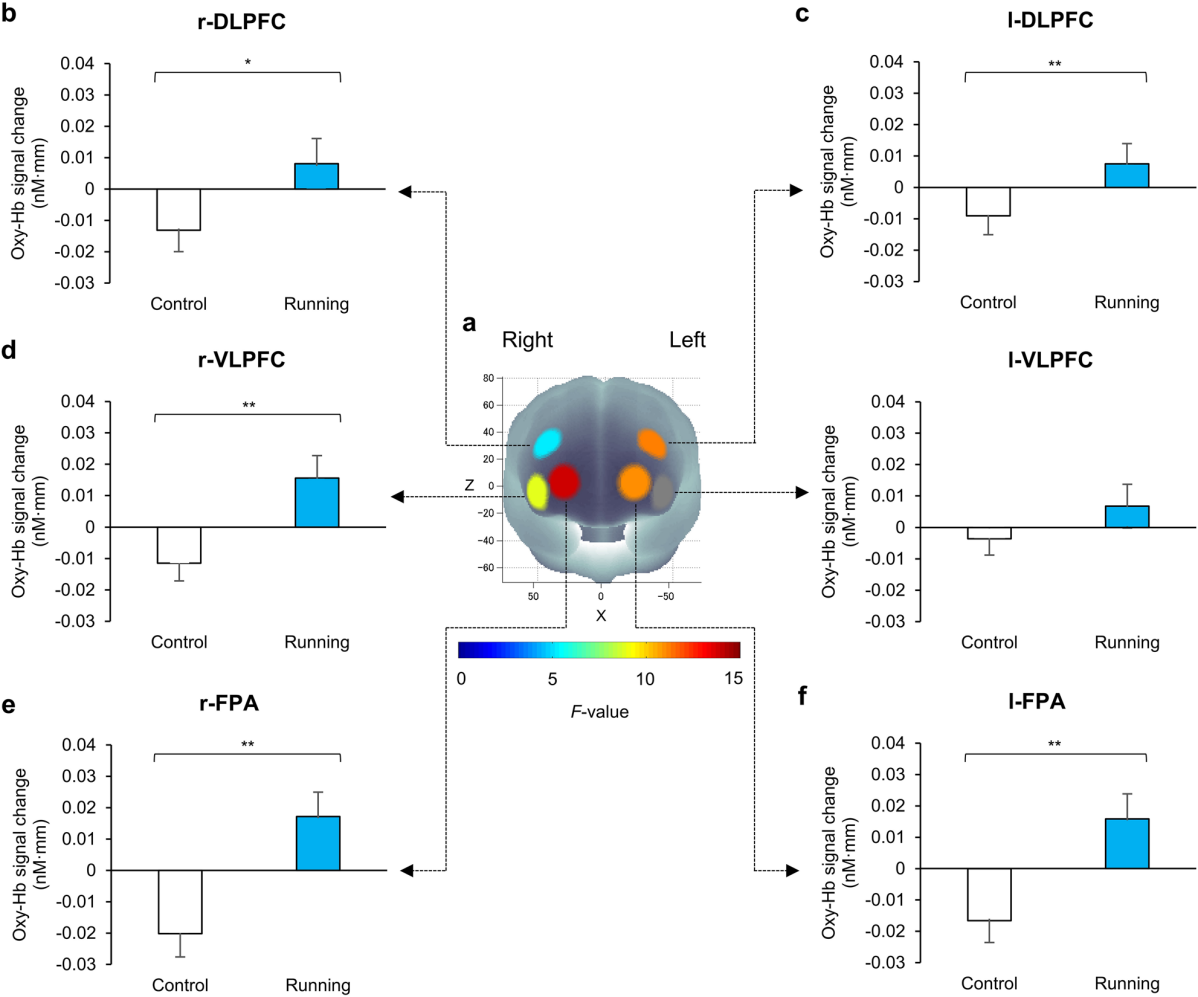
Running elicits prefrontal activation in response to the Stroop interference difference ([incongruent—neutral of post-session]—[incongruent—neutral of pre-session]). (**a**) *F*-map of oxygenated hemoglobin (Oxy-Hb) signal change, *F*-values are indicated as in the color bar. Among the 6 regions of interest (ROIs), significant differences are found in (**b**) the right dorsolateral prefrontal cortex, (**c**) the left dorsolateral prefrontal cortex, (**d**) the right ventrolateral prefrontal cortex, (**e**) the right frontopolar area and (**f**) the left frontopolar area. Data are mean ± SE, **p < 0.01, *p < 0.05.

There were no significant differences in pre-sessions of Stroop-interference-related cortical activation between CON and RUN conditions in any ROIs. The pre-session data for the CON and RUN sessions, which were free from any effect of exercise, were averaged and served as substrates for a ROI-wise analysis. Significant Stroop-interference-related cortical activations were found in all ROIs (*p* < 0.05, one-sample *t*-test, FDR correction) indicating that the Stroop interference effect could be observed in this study (Fig. 3). The effect of running on prefrontal activation in all ROIs was subsequently determined and the results revealed that Stroop-interference-related cortical activations had significant interaction between session (CON, RUN) and time (pre, post) in 5 ROIs: l-DLPFC (*F*(1, 25) = 11.26, *p* = 0.003), l-FPA (*F*(1, 25) = 11.04, *p* = 0.003), r-DLPFC (*F*(1, 25) = 5.36, *p* = 0.029), r-VLPFC (*F*(1, 25) = 8.98, *p* = 0.006) and r- FPA (*F*(1, 25) = 13.85, *p* = 0.001) (repeated-measures two-way ANOVA). Finally, changes in oxy-Hb with Stroop interference in these 5 ROIs were examined and the results revealed that the RUN session had a significantly greater increase of oxy-Hb with Stroop interference than did the CON session in all 5 ROIs: l-DLPFC (*t*(25) = 3.36, *p* = 0.003, Cohen’s *d* = 0.66), l-FPA (*t*(25) = 3.32, *p* = 0.003, Cohen’s *d* = 0.65), r-DLPFC (*t*(25) = 2.31, *p* = 0.029, Cohen’s *d* = 0.45), r- VLPFC (*t*(25) = 3.00, *p* = 0.006, Cohen’s *d* = 0.59) and r-FPA (*t*(25) = 3.72, *p* = 0.001, Cohen’s *d* = 0.66) (paired *t*-test, FDR correction; Fig. 4). Cortical activation patterns during performance of the CWST in the post-session of RUN were provided in Supplementary B.

### The association among mood, behavior and fNIRS results

Significant coincidences between running-induce mood change and running-effected behavioral Stroop performance were observed. Increased arousal and pleasure levels significantly coincided with shortened Stroop interference RT (χ^2^mc (1,26) = 17.05, *p* < 0.001 and χ^2^mc (1,26) = 14.06, *p* < 0.001, respectively; McNemar test). Enhanced arousal levels were consistently found to significantly coincide with all 5 ROIs (l-DLPFC, χ^2^mc (1,26) = 14.06, *p* < 0.001; l-FPA, χ^2^mc (1,26) = 18.05, *p* < 0.001; r-DLPFC, χ^2^mc (1,26) = 16.06, *p* < 0.001; r-VLPFC, χ^2^mc (1,26) = 15.06, *p* < 0.001 and r-FPA, χ^2^mc (1,26) = 17.05, *p* < 0.001; McNemar test) as were pleasure levels (l-DLPFC, χ^2^mc (1,26) = 7.56, *p* = 0.004; l-FPA, χ^2^mc (1,26) = 14.06, *p* < 0.001; r-DLPFC, χ^2^mc (1,26) = 12.07, *p* < 0.001; r-VLPFC, χ^2^mc (1,26) = 9.60, *p* = 0.001 and r-FPA, χ^2^mc (1,26) = 13.07, *p* < 0.001; McNemar test) (Table 1). In addition, shortened Stroop interference RT demonstrated significant coincidence with all 5 ROIs (l-DLPFC, χ^2^mc (1,26) = 7.69, *p* = 0.003; l-FPA, χ^2^mc (1,26) = 11.53, *p* < 0.001; r-DLPFC, χ^2^mc (1,26) = 9.60, *p* = 0.001; r-VLPFC, χ^2^mc (1,26) = 6.72, *p* = 0.008 and r-FPA, χ^2^mc (1,26) = 10.56, *p* = 0.001; McNemar test).

**Table 1:** Coincidence frequency between running-induced mood change and sub-regional prefrontal activation for McNemar test.

		Oxy-Hb in l-DLPFC		Total	Oxy-Hb in l-FPA		Total	Oxy-Hb in r-DLPFC		Total	Oxy-Hb in r-VLPFC		Total	Oxy-Hb in r-FPA		Total
		−	+		−	+		−	+		−	+		−	+	
Arousal	+	9	16	25	5	20	25	7	18	25	8	17	25	6	19	25
	−	0	1	1	0	1	1	0	1	1	0	1	1	0	1	1
	Total	9	17	26	5	21	26	7	19	26	8	18	26	6	20	26
Pleasure	+	7	14	21	5	16	21	7	14	21	7	14	21	6	15	21
	−	2	3	5	0	5	5	0	5	5	1	4	5	0	5	5
	Total	9	17	26	5	21	26	7	19	26	8	18	26	6	20	26

Presented values are number of participants. The contrasts between [(post-session)−(pre-session)] in RUN and [(post-session)−(pre-session)] in CON for each variable was subjected to analysis. Significant coincidences among variables were observed. l-DLPFC = left dorsolateral prefrontal cortex, l-FPA = left frontopolar area, r-DLPFC = right dorsolateral prefrontal cortex, r-VLPFC = right ventrolateral prefrontal cortex, r-FPA = right frontopolar area.

## Discussion

Here we used fNIRS to test the hypothesis that acute running stimulates the PFC, which results in increasing positive mood and executive function. We found that a single bout of moderate-intensity running at 50%${\dot{\text{V}}\text{O}}_{{{\text{2peak}}}}$, the most popular running condition, enhanced mood not only through arousal but also through pleasure and also influenced executive function, as evidenced in the shortening of Stroop interference RT, while activating the cortical areas associated with cognition and mood regulation. These results clearly support the hypothesis that acute moderate running benefits mood and executive function coincident with cortical activation in the prefrontal subregions involved in mood regulation. Referred to our previous evidence that suggested single benefit of moderate pedaling exercise in enhancing cognition without reporting pleasant mood change based-on the TDMS, the current study could demonstrate dual benefits of moderate running exercise in increasing cognition and pleasure level, which may influence the brain in regulating stress, leading to mental health^4,5,43,46–48^.

Running apparently enhanced arousal levels compared to a resting control, and this is consistent with our previous studies^4,5^. Moreover, we also revealed that 10 min of moderate-intensity running benefited pleasure levels, which also agrees with previous evidence reporting increased positive affection after a single bout of moderate-intensity running^49,50^. Based on these findings, running may be considered an exercise mode that benefits mood, which is an important factor for exercise adherence^51^. Another psychological response observed here is an observed RPE that was lower than the reference range^45^. Theoretically, RPE is a recognized marker of intensity and homeostatic disturbance during exercise as well as an effective way to monitor physical effort^52,53^. In regulating RPE, we propose that the prefrontal cortex may be involved in the interpretation of physiological signals, which are integrated between internal and external factors present in exercise environments to determine exercise tolerance^52,54^. Therefore, running seems to have the potential to influence prefrontal regions to diminish perceived physical effort (lower RPE) that presumably benefits exercise performance. However, this account remains somewhat speculative.

The behavioral response investigated using the CWST revealed longer RT with higher ER in the incongruent condition compared to the neutral condition, demonstrating that the Stroop interference effect could be observed across all sessions. The Stroop interference time was subsequently determined, and the results support our hypothesis that running shortened Stroop interference time compared to the resting control^3–5^. Moreover, significant coincidence between enhanced mood (increased arousal and pleasure levels) and shortened Stroop interference time was revealed. Together, these results allow us to postulate that a running-induced positive mood could enhance behavioral Stroop performance resulting in increased executive function, although the directionality of this relationship cannot be directly inferred from our data^34,37^.

**Figure 5:**
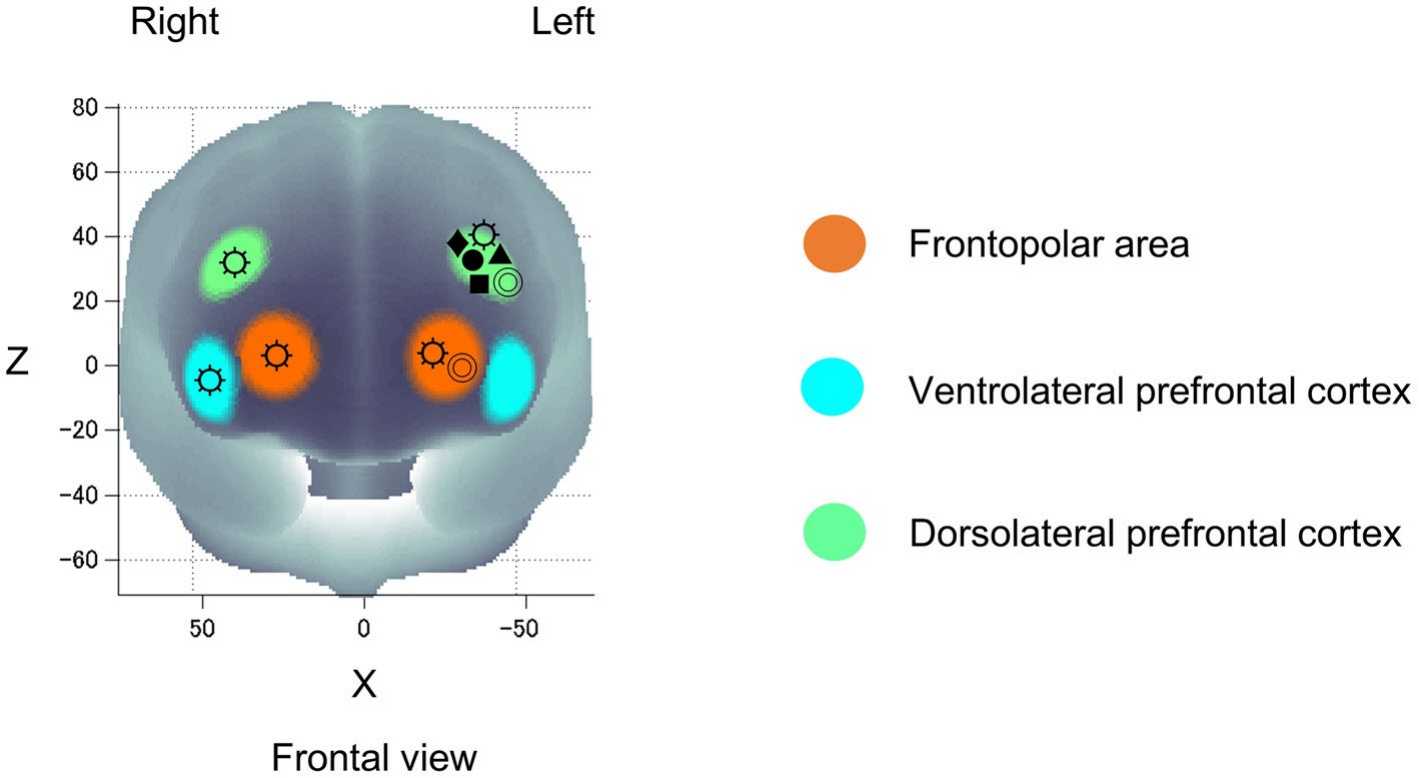
Exercise elicits prefrontal activation in response to Stroop interference difference ([incongruent—neutral of post-session]—[incongruent—neutral of pre-session]) of the present moderate-intensity running study (☼) compared to previous studies with pedaling exercise: moderate-intensity exercise (Yanagisawa et al., 2010: ●), moderate-intensity exercise (Endo et al., 2013: ■), mild-intensity exercise (Byun et al., 2014: ◎), high-intensity intermittent exercise (Kujach et al., 2018: ♦) and moderate-intensity exercise with music (Suwabe et al., 2021: ▲). All studies measured cortical activation using fNIRS while participants performed the color-word matching Stroop task (CWST).

The prefrontal cortex – the neural substrate underpinning the performance of the CWST – revealed significant increases of Oxy-Hb in response to Stroop interference for all ROIs, showing that the Stroop interference effect could be observed as in our previous studies^4,5^. The effect of running on prefrontal activation was subsequently determined, and the results showed that running led to significant increases in activation in all ROIs except the l-VLPFC. The l-DLPFC, which plays an important role in the implementation of cognitive control, had increase of Oxy-Hb response to Stroop interference, which corresponds to the results of previous studies including our own^4,5,30,55,56^. Besides, the l-DLPFC was reported to implicate in mood regulation in mood disorders, demonstrating significant coincidence between running-induced positive mood and l-DLPFC activation as we hypothesized^19–21^. The right DLPFC, which is involved in conflict resolution (faster reaction time after incongruent trials) and Stroop accuracy, was also showed significant activation^31,57,58^. The right-VLPFC, which is associated with mood and emotion regulation, was observed to have significant activation coinciding with increased arousal and pleasure levels^19–21,59^. In addition, the FPA, a brain region which generally activates with the DLPFC to response to tasks involving manipulation and monitoring, such as planning for action, was observed to have significant both sides activation^60^. It is considered that running-induced bilateral prefrontal activation, differing from the previous studies using pedaling exercise that revealed prominent l-DLPFC activation (Fig. 5)^3–5,43,61^. Taken together, these results support our hypothesis that an acute bout of moderate-intensity running elicits mood improvement and enhances executive function coinciding with prefrontal subregion activations involved in mood regulation.

The neural mechanisms for running-elicited cortical activation have remained unclear. Running requires top-down feedforward control responses to multi-modal sensory information that partially involve the prefrontal cortex to execute coordinated movement and balance^13–15,17,18^. Specific features of running, such as foot strike, may benefit the brain activation by enhancing blood flow velocity in the middle cerebral artery^24^. However, the mechanism of this finding still unclear. Besides, a mechanical force from vertical head acceleration, may induce serotonin receptor internalization in the prefrontal cortex, influencing emotion and cognitive control based-on animal study^25^. With respect to the theoretical concept of acute exercise-enhanced cognition, it is considered that running has the potential to induce neural activation by activating the RAS to enhance arousal levels, and by projecting neuronal signals to the prefrontal cortex^32,33,37–39^. This neuronal response is mostly modulated by three neurotransmitter systems: the noradrenergic, dopaminergic and serotoninergic systems, which originate from the brainstem and project profusely throughout the forebrain, playing a role in cognition and mood regulation^34–39^.

That insufficient physical activity leads to physical and mental illness has been recognized as a global issue. It is important to demonstrate a minimal effective exercise that will benefit both mental and physical health. We have found that even a 10-min single bout of moderate-intensity running can boost both mood and executive function; in particular, broad prefrontal activation as well as enhanced pleasure levels are important for exercise adherence. These fruitful findings are meaningful in terms of health promotion because running is considered a practical exercise and is one of the most popular recreation activities worldwide.

Here we investigated only the effect of running, but not a pedaling because of the following methodological limitations. First, to gain statistical robustness through the process of neuroimaging analysis using fNIRS, we must limit session numbers. Second, we have already found the exact findings on pedaling using the same experimental protocol for ensuring the specificity of running effects^4,5,30^. Indeed, we successfully revealed the dual effects of running boosting mood and executive function, different from those pedaling (Fig. 5).

## Conclusions

The current study reveals that a 10-min single-bout of moderate-intensity running elicits a positive mood and increased executive function by enhancing arousal levels coincidentally with activation in prefrontal subregions involved in mood regulation. We provide evidence for the neural substrates behind mood enhancement and increased executive function induced by acute moderate-intensity running. To this end, these finding are valuable in supporting moderate running effect on mental health since running is an easily accessible form of exercise requiring minimal equipment and sport structure. This should shed light on the specificity of running among a varieties of exercise prescriptions promoting mental health.

## Materials and methods

### Participants

Twenty-six young adults participated in this study. All participants were Japanese native speakers, right-hand dominant, with normal, or corrected-to-normal vision and normal color vision. No participant reported a history of neurological or psychiatric disorders, or had a disease requiring medical care. The characteristics of participants are presented in Table 2. This study was approved by the Institutional Ethics Committee of the University of Tsukuba, and the protocol was in accordance with the latest version of the Helsinki Declaration. Complete information about the study was given to the participants prior to obtaining written informed consent from all of them.

**Table 2:** The characteristics of participants (n = 26).

	Male (n = 18)	Female (n = 8)
Age (yr)	23.22 ± 2.24	22.88 ± 1.96
Weight (kg)	67.78 ± 8.33	55.25 ± 10.23
Height (cm)	172.89 ± 3.87	158.66 ± 5.39
BMI (kg/m^2^)	22.66 ± 2.59	21.80 ± 2.79
${\dot{\text{V}}\text{O}}_{{{\text{2peak}}}}$ (ml/kg/min)	53.05 ± 7.62	39.79 ± 5.63
HR_max_ (bpm)	191.06 ± 10.42	194.88 ± 5.36

Presented values are mean ± SD. BMI = Body Mass Index; ${\dot{\text{V}}\text{O}}_{{{\text{2peak}}}}$ = Peak oxygen uptake; HR_max_ = Maximal Heart Rate.

### Experimental procedure

**Figure 6:**
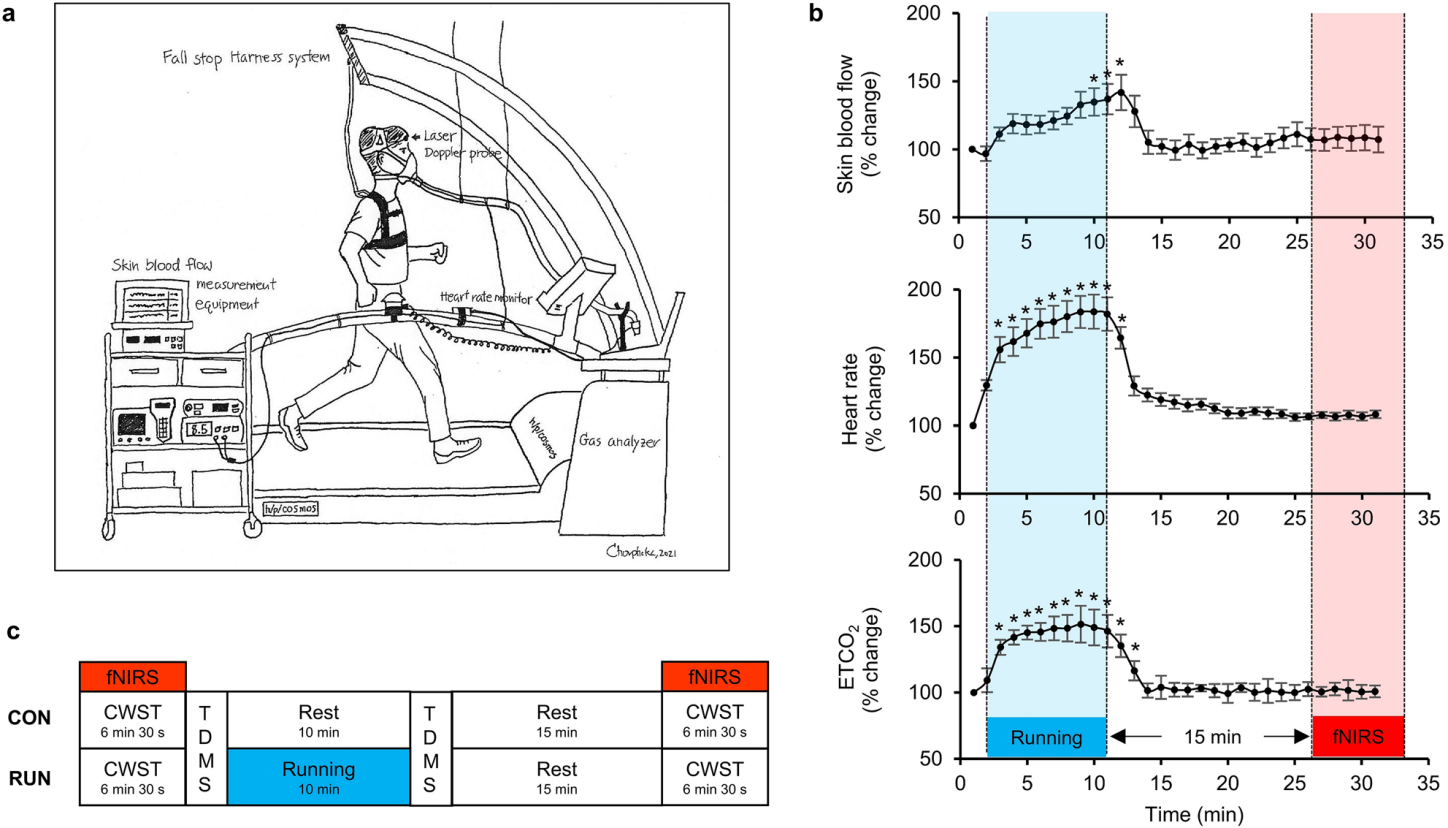
(**a**) Illustration of experimental setup for the second preliminary step: non-cortical physiological measurements. A laser Doppler probe was attached to the forehead to assess skin blood flow. (**b**) Results of non-cortical physiological parameters at baseline (set at 100%), during 10 min of moderate-intensity running and 20 min rest. Time points with a significant change compared to baseline are identified with asterisks (*p* < 0.05, one-way ANOVA with Dunnett correction). All physiological variables fully returned to baseline within 15 min. Thus, fNIRS measurement was started 15 min after running to eliminate possible signal contamination. ETCO_2_ = End-tidal carbon dioxide; (**c**) Experimental design for control (CON) and running (RUN) sessions. CWST = color-word matching Stroop task, TDMS = Two-Dimensional Mood Scale, fNIRS = Functional near-infrared spectroscopy.

The experimental procedure consisted of three major steps. First, we measured maximal oxygen uptake (${\dot{\text{V}}\text{O}}_{{{\text{2peak}}}}$) to determine the appropriate individual intensity of moderate exercise, which is 50%${\dot{\text{V}}\text{O}}_{{{\text{2peak}}}}$ based on the definition of exercise intensity of the American College of Sports Medicine^45^. Second, we investigated non-cortical physiological signals induced by running in 6 participants, such as skin blood flow, to specify proper fNIRS measurement times after running in order to eliminate possible contamination of the fNIRS signal^62^. We found that all physiological variables fully returned to the baseline within 15 min. Therefore, 15 min after running, we started fNIRS measurements to avoid possible signal contamination (Fig. 6a and b). Details of the two preliminary steps are provided in Supplementary C. Finally, we determined the effect of a single bout of moderate-intensity running on mood and executive function. Two sessions, control (CON) and running (RUN), were randomly conducted with a counterbalance measure design on different days. In the RUN session, fNIRS was used to measure prefrontal hemodynamic changes while performing the CWST before and 15 min after running to avoid non-cortical physiological signal contamination. Participants ran for 10 min on a treadmill at a personalized speed. Mood state was assessed before and after running. In the CON session, participants rested for 10 min instead of running (Fig. 6c).

### Psychological measures

Psychological mood state was measured before and after running using the Two-Dimensional Mood Scale (TDMS)^63^. The TDMS is a momentary mood scale that consists of eight words describing arousal and pleasure states (energetic, lively, lethargic, listless, relaxed, calm, irritated and nervous). The participants were asked to indicate how they were feeling for each mood-expressing word using a 6-point Likert scale that ranged from 0 = Not at all to 5 = Extremely. Then, arousal and pleasure levels were calculated.

### Behavioral measures

**Figure 7:**
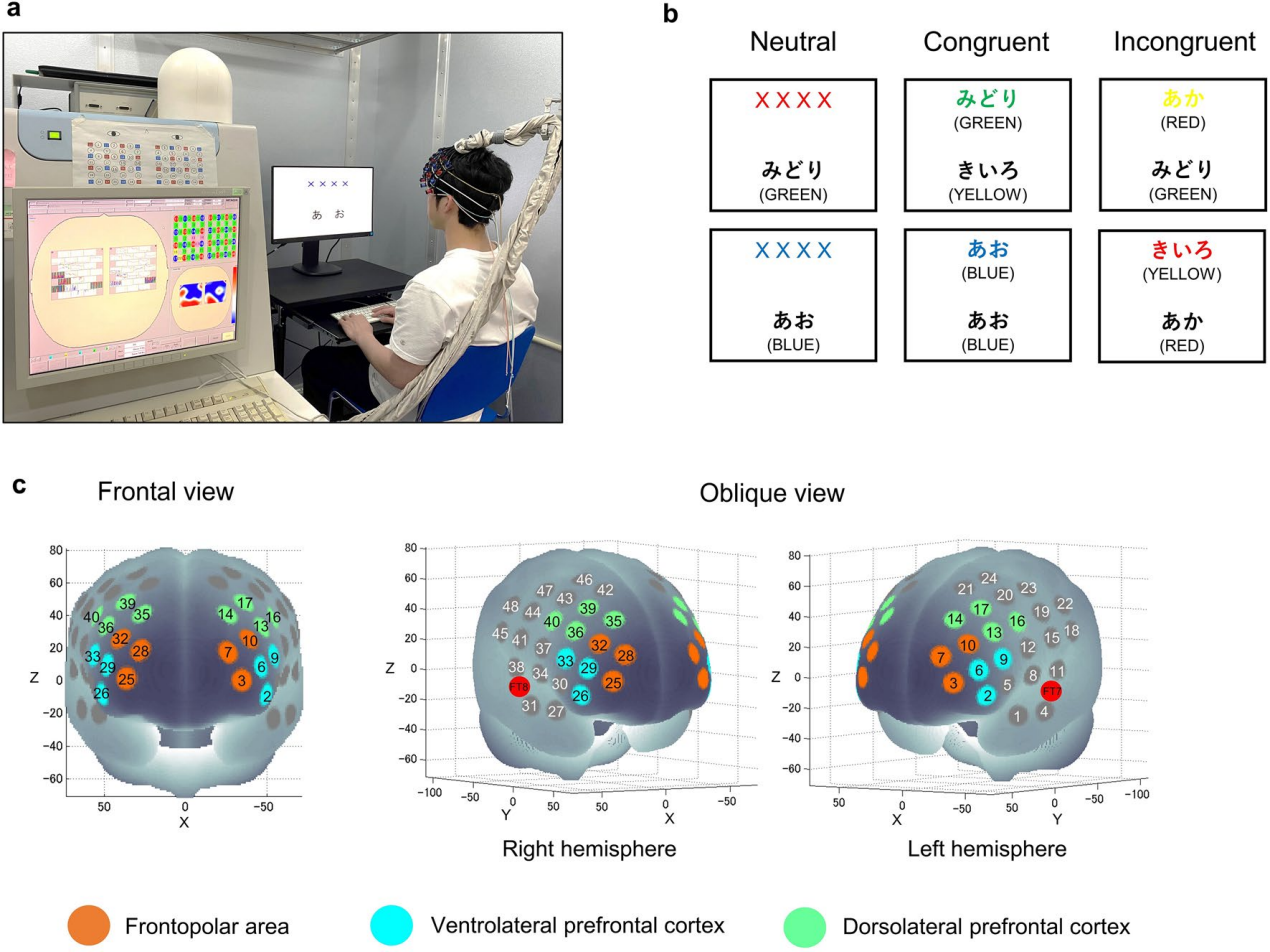
(**a**) Illustration of experimental setup for fNIRS measurements during the color-word matching Stroop task (CWST). (**b**) Examples of the CWST neutral, congruent and incongruent conditions. The presented words were written in Japanese. English translations are shown in parentheses. (**c**) Spatial profiles of fNIRS channels used in the present study. Two sets of probe holders were placed to cover both lateral prefrontal activation foci as in our previous studies. Colors indicate each region of interest (ROI) in the lateral part of the prefrontal cortex.

Executive function was investigated using an event-related version of the CWST^3,4,43,64^. The test appeared on the screen with 2 rows of letters (Fig. 7a). The participants were instructed to decide whether the color of the letters in upper row corresponded to the color name in the lower row. They were also asked to place their index fingers on “yes” and “no” buttons and respond to the test by pressing the appropriate button as fast as they could. Subsequently, reaction time (RT) and error rate (ER) were calculated. The CWST consisted of three conditions: neutral, congruent and incongruent. For the neutral condition, the upper row was presented as a row of X’s (XXXX) printed in red, green, blue or yellow, and the lower row presented the word ‘RED’, ‘GREEN’, ‘BLUE’ or ‘YELLOW’ printed in black. For the congruent condition, the upper row contained the word ‘RED’, ‘GREEN’, ‘BLUE’ or ‘YELLOW’ printed in the congruent color (e.g., ‘RED’ was printed in red), and the lower row presented the same words as in the lower row of the neutral condition. For the incongruent condition, the upper row presented the color word printed in an incongruent color to produce interference between the color word and the color name (e.g., ‘RED’ was printed in green), and the lower row presented the same words as in the lower row of the neutral and congruent conditions (Fig. 7b). Each experimental session consisted of 30 trials, made up of 10 neutral, 10 congruent and 10 incongruent trials, which appeared in random order. The upper row was presented 100 ms before the lower row in order to shift visual attention. The trial remained on the screen until the participant responded or for 2 s, whichever was shorter. Then, a fixation cross appeared on the screen as an inter-stimulus interval for 10–12 s to avoid prediction of the timing of the subsequent trial. Stroop interference, an index of executive function in the prefrontal cortex, was calculated as the difference in reaction time between the incongruent and neutral conditions^30^. All words were written in Japanese. The participants performed three practice sessions to ensure that they understood and were familiarized with the CWST well before starting the experiment.

### fNIRS data acquisition

Cortical hemodynamic changes were monitored using the multichannel fNIRS optical topography system ETG-7000 (Hitachi Medical Corporation, Japan) and adopting two wavelengths of near-infrared light (785 and 830 nm). The optical data from fNIRS was analyzed based on the modified Beer-Lambert Law as previously described^65,66^. With this method, we were able to calculate signals reflecting the oxygenated hemoglobin (oxy-Hb) concentration changes in a unit of millimolar-millimeter (mM∙mm)^66^. The sampling rate was set at 10 Hz. For fNIRS probe placement, two sets of 4 × 4 multichannel probe holders, which consist of 8 illuminative and 8 detective probes arranged alternately at an inter-probe distance of 3 cm resulting in 24 channels (CH) per set, were placed to cover both lateral prefrontal activation foci as in previous studies^3,4^. The left probe holder was placed such that probe 5 (between CH4 and CH11) was placed over FT7, with the medial edge of the probe column parallel to the medial line. Likewise, the right probe holder was symmetrically placed on the left hemisphere (Fig. 7c). We adopted a virtual registration method to register fNIRS data to Montreal Neurological Institute (MNI) standard brain space^67,68^. With this method, we were able to place a virtual probe holder on the scalp by stimulating the holder’s deformation and by registering probes and channels onto a reference brain in the magnetic resonance image (MRI) database^69,70^. We probabilistically estimated the MNI coordinate values for fNIRS in order to obtain the most likely microanatomical prediction for locations of the given channels as well as the spatial variability of the estimation^71^. Finally, a MATLAB function was used to label the estimated locations in a macro-anatomical brain atlas^72^.

### Analysis of fNIRS data

Prefrontal Oxy-Hb changes that occurred while performing the CWST were calculated as shown in our previous studies^3,4,40–43^. Individual timeline data for each channel were preprocessed with a band-pass filter using a high-pass filter (0.04 Hz) to remove baseline drift and a low-pass filter (0.30 Hz) to screen out heartbeat pulsations. Then, channel-wise and subject-wise contrasts were calculated by the inter-trial mean of differences between peak (4–11 s after trial onset) and baseline (0–2 s before trial onset) periods. The contrasts obtained were subsequently subjected to a second level of random effects group analysis. This study adopted LBPA40, a widely used method among anatomical labeling systems, to combine 3—4 neighboring channels to form each region of interest (ROI)^72^. The regions included the DLPFC (l-DLPFC: channels 13, 14, 16 and 17; r-DLPFC: channels 35, 36, 39 and 40), the VLPFC (l-VLPFC: channels 2, 6 and 9; r-VLPFC: channels 26, 29 and 33) and the FPA (l-FPA: channels 3, 7 and 10; r-FPA: channels 25, 28 and 32). LBPA40 is considered valid because optical properties of neighboring channels are known to be similar^73^. However, with this method, optical properties in different ROIs can cause systematic bias during statistical analysis. Therefore, we limited analyses to ROI-wise and used a false discovery rate (FDR) to control the low proportion of false positives^74^.

### Statistical analysis

Psychological mood state was subjected to repeated-measures two-way ANOVA with session (CON, RUN) and time (pre, post) as within-subject factors. Bonferroni’s post hoc test was subsequently processed after a significant F value was observed. Regarding behavioral Stroop performance, RT and ER were first subjected to repeated-measures three-way ANOVA with condition (neutral, incongruent), session (CON, RUN) and time (pre, post) as within-subject factors to examine whether the general tendencies for the Stroop task could be reproduced in all conditions. Then, the effect of running on Stroop task performance was analyzed by repeated-measures two-way ANOVA with session (CON, RUN) and time (pre, post) as within-subject factors followed by Bonferroni’s post hoc test. For the fNIRS data, pre-sessions of cortical activation for CON and RUN sessions, which were free from any effect of running, were first examined to determine whether the Stroop interference effect (the difference in oxy-Hb between incongruent and neutral conditions) could be observed. The Stroop interference levels for the CON and RUN sessions were averaged and served as substrates for a ROI-wise analysis with FDR for controlling the low proportion of false positives. Only significant ROIs for Stroop interference were subsequently analyzed for the effect of running on prefrontal activation using repeated-measures two-way ANOVA with session (CON, RUN) and time (pre, post) as within-subject factors followed by FDR correction. This study adopted the McNemar test to assess the association among running-influenced mood change, running-effected behavioral Stroop performance and running-elicited cortical activation in a binomial manner. The contrasts between [(post-session)—(pre-session)] in RUN and [(post-session)—(pre-session)] in CON for each variable was subjected to the McNemar test. Details of the McNemar test are provided in Supplementary D. The statistical significance level was set a priori at *p* < 0.05. SPSS Statistical Packages version 24 (SPSS, Inc., USA) was used for statistical analysis.

## Supplementary Information

Supplementary Information.

## Data Availability

All data that support the findings of this study are available from the corresponding author by request with no restrictions.
