# Supplementary material for: Benefit of human moderate running boosting mood and executive function coinciding with bilateral prefrontal activation
Source: Sci Rep. 2021 Nov 22;11:22657. doi: 10.1038/s41598-021-01654-z (PMC8608901; doi:10.1038/s41598-021-01654-z)
Supplement: Supplementary file 1 — Supplementary Information. [file 41598_2021_1654_MOESM1_ESM.pdf]

Supplementary Materials

Benefit of human moderate running boosting mood and executive function coinciding with bilateral prefrontal activation

Chorphaka Damrongthai[1,2,3], Ryuta Kuwamizu[1], Kazuya Suwabe[2,4], Genta Ochi[5], Yudai Yamazaki[2], Takemune Fukuie[6], Kazutaka Adachi[7], Michael A. Yassa[2,8,9], Worachat Churdchomjan[3], Hideaki Soya[1,2,*]

[1]Laboratory of Exercise Biochemistry and Neuroendocrinology, Faculty of Health and Sport Sciences, University of Tsukuba, Ibaraki 305-8574, Japan
[2]Advanced Research Initiative for Human High Performance (ARIHHP), Faculty of Health and Sport Sciences, University of Tsukuba, Ibaraki 305-8574, Japan
[3]Faculty of Physical Therapy and Sport Medicine, Rangsit University, Pathum Thani 12000 Thailand
[4]Faculty of Health and Sport Sciences, Ryutsu Keizai University, Ryugasaki 301-8555 Japan
[5]Department of Health and Sports, Niigata University of Health and Welfare, Niigata 950-3198 Japan
[6]School of Nursing and Social Services, Health Sciences University of Hokkaido, Hokkaido 061-0293 Japan
[7]Laboratory of Applied Anatomy, Faculty of Health and Sport Sciences, University of Tsukuba, Ibaraki 305-8574, Japan
[8]Department of Neurobiology and Behavior, University of California, Irvine, CA 92679-3800, USA
[9]Center for the Neurobiology of Learning and Memory, University of California, Irvine, CA 92679-3800, USA

***Correspondence to**: Hideaki Soya, Ph.D.
Sports Neuroscience Division, Department of Mind, Advanced Research Initiative for Human High Performance (ARIHHP), Laboratory of Exercise Biochemistry and Neuroendocrinology, Faculty of Health and Sport Sciences, University of Tsukuba, 1-1-1 Tennoudai, Tsukuba, Ibaraki 305-8574, Japan; Telephone/Fax: +81-29-853-2620
E-mail: soya.hideaki.gt@u.tsukuba.ac.jp

Supplementary A

A comparison of baseline mood state between CON and RUN

Consider the baseline values, there was no significant difference in arousal level and pleasure level between groups ($t(25) = 0.48$, $p = 0.638$ and $t(25) = 1.47$, $p = 0.155$, respectively; paired *t*-test) as shown in Fig. 1.

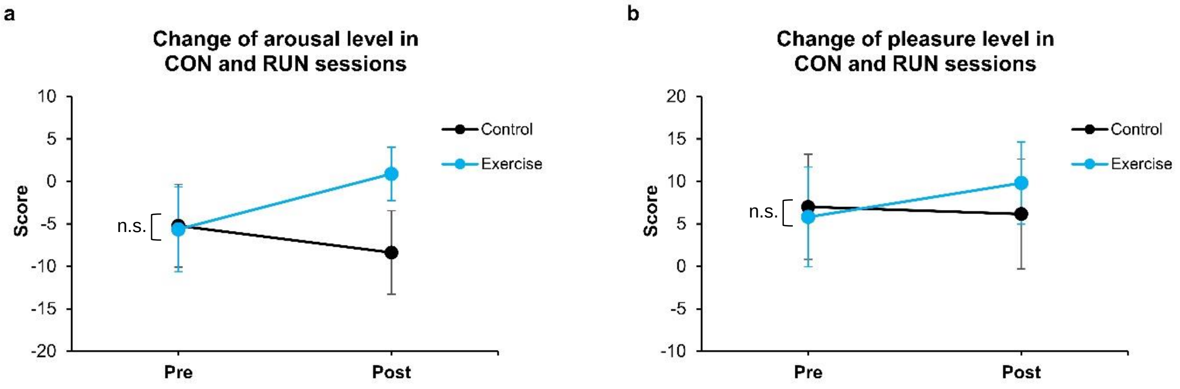

Fig. 1. Demonstrate change of mood state between CON and RUN sessions: (a) arousal level and (b) pleasure level. n.s. = non-significant different

Supplementary B

B.1. Cortical activation patterns in response to the CWST

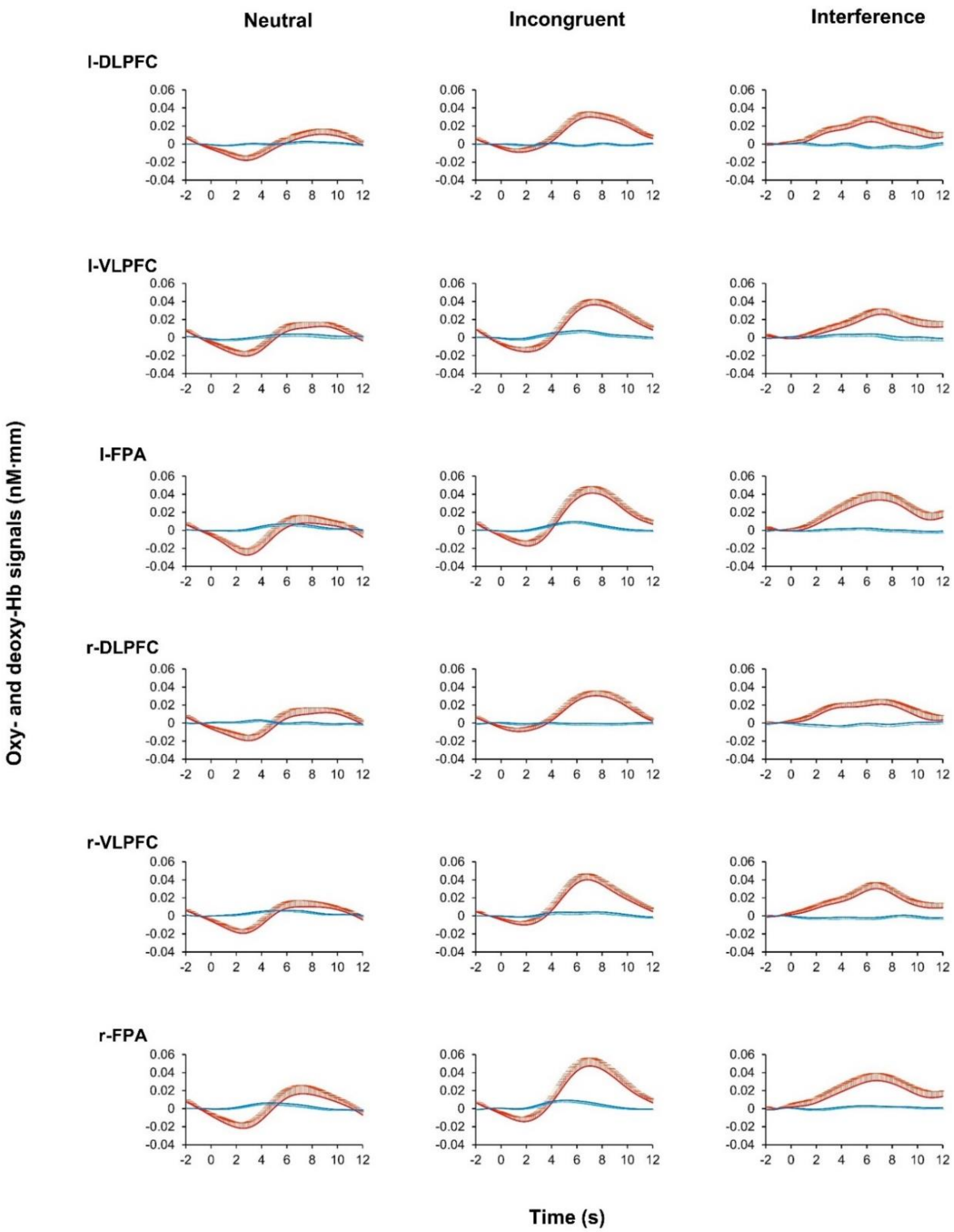

Fig 2. Cortical activation pattern during performed the color-word Stroop task (CWST) in the post-session of exercise, demonstrating in the 6 regions of interest (ROIs). Red and blue line indicated Oxy- and deoxy-Hb signals respectively.

B.2. Cortical activation in response to the Stroop interference

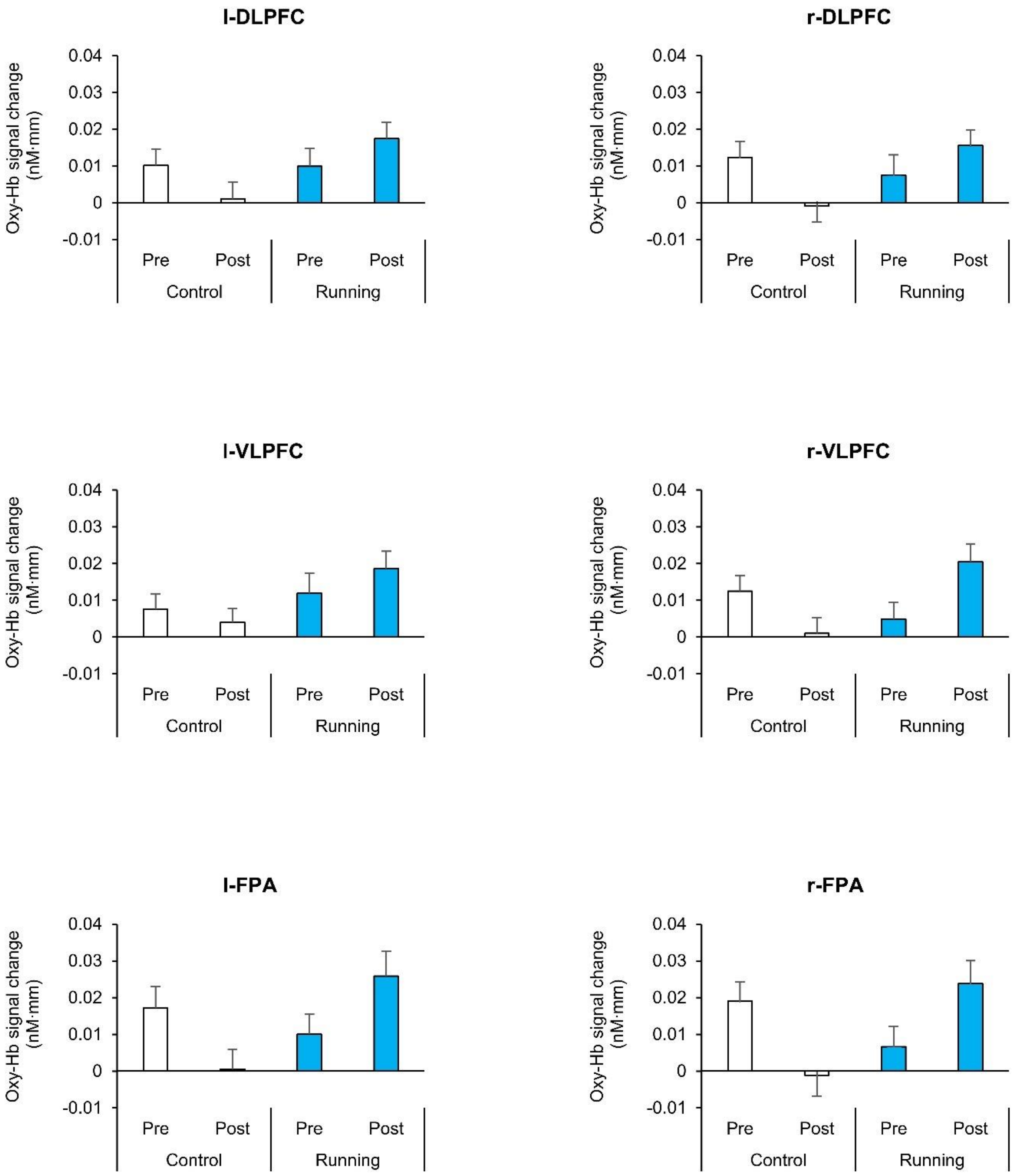

Fig 3. Prefrontal activation in response to the Stroop interference [incongruent - neutral], demonstrating in the 6 regions of interest (ROIs).

Supplementary C

C.1. $\dot{V}O_{2peak}$ determination

Maximal oxygen uptake ($\dot{V}O_{2peak}$) was firstly determined to indicate appropriate individual intensity of moderate exercise that was 50%$\dot{V}O_{2peak}$ based on the definition of exercise intensity of the American College of Sports Medicine. [1]

A treadmill (Pulsar® h/p/cosmos, Germany) was used for incremental exercise test. The test began with 2 min resting and 3 min warm-up at 5 km/hr (female: 4 km/hr). Then, a treadmill speed was increased 1 km/hr every minute with maintaining treadmill slope at 0.1% until the subject reached volitional exhaustion, and continuously follow by 3 min cool-down to complete the test.[2] Heart rate (HR) and rate of perceived exertion (RPE) were recorded every minute.[3] Gas analyzer (Aeromonitor AE300, Minato Medical Science, Japan) was used for measuring ventilation parameters those were oxygen consumption ($\dot{V}O_2$), Carbon dioxide production ($\dot{V}CO_2$) and respiratory exchange ratio (R) with sampling rate 0.1 Hz.[4] $\dot{V}O_{2peak}$ was determined if the subject met 2 of the following criterias: R > 1.15, ± 10 bpm of age predicted maximal HR or RPE = 19-20.[5] Physiological parameters and treadmill speed at volitional exhaustion were shown in Table 1.

Table 1. Physiological parameters and treadmill speed at volitional exhaustion (n=26).

	**Male (n=18)**	**Female (n=8)**
HR (bpm)	191.06 ± 10.42	194.88 ± 5.36
RPE	19.44 ± 0.62	19.00 ± 0.93
R	1.11 ± 0.04	1.12 ± 0.03
$\dot{V}O_{2peak}$ (ml/kg/min)	53.05 ± 7.62	39.79 ± 5.63
Treadmill speed (km/hr)	17.61 ± 2.35	13.88 ± 1.64

Values are mean ± SD, HR = heart rate, RPE = rate of perceived exertion, R = respiratory exchange ratio, $\dot{V}O_{2peak}$ = maximal oxygen uptake

To define exertion needed to achieve 50%$\dot{V}O_{2peak}$, $\dot{V}O_2$ (x-coordinate) was plotted against the treadmill speed (y-coordinate). Then, the treadmill speed at 50%$\dot{V}O_{2peak}$ was determined by a simple linear regression formula that calculated from Microsoft Excel.[6] This calculated treadmill speed was assigned as the exertion during 10 min running.

C.2. Investigation of non-cortical physiological changes induced by exercise.

Exercise induced whole-body physiological changes. To measure cortical hemodynamic after exercise by using Functional near-infrared spectroscopy (fNIRS), it was necessary to determine how did non-cortical physiological parameters such as skin blood flow (SBF) and HR change to specify proper fNIRS measurement time after exercise to eliminate possible signal contamination.[7]

A laser-Doppler probe (FLO-C1, OMEGAWAVE, Japan) was attached at Fpz of the international 10-20 system to assess SBF. End-tidal carbon dioxide ($ETCO_2$) which could indicate cardiac output and pulmonary blood flow was measured by gas analyzer (Aeromonitor AE300, Minato Medical Science, Japan).[8] The experiment protocol started with 3 min rest, continuing with 10 min moderate-intensity running and rest again for 20 min. SBF, HR, $\dot{V}O_2$ and $ETCO_2$ were monitored every minute through the experiment. Six Japanese young adults (2 females) participated in the study: mean age 23.67 $\pm$ 2.25 years [range 22-28], weight 59.68 $\pm$ 7.64 kg, height 164.66 $\pm$ 5.46 cm, $\dot{V}O_{2peak}$ 48.16 $\pm$ 8.64 ml/kg/min. Non-cortical physiological change induced by exercise were shown as Fig 1.

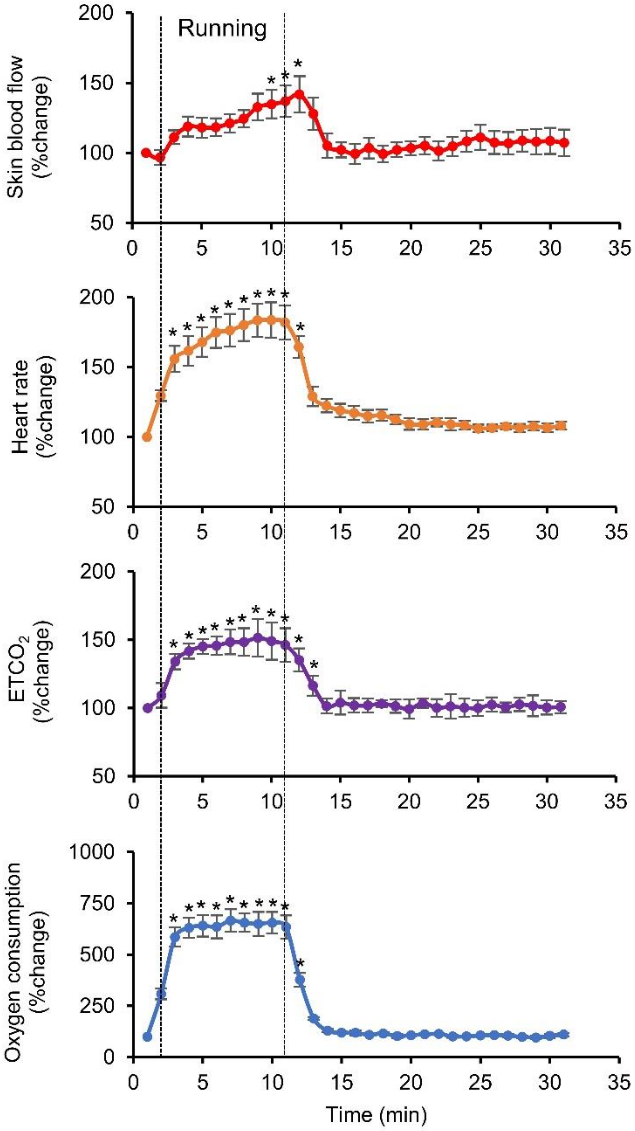

Fig 4. Illustrations of the non-cortical physiological parameters at baseline (set at 100%), during 10 min moderate-intensity running and 20 min rest. Inter-subject mean at each time point were plotted with error bar indicated by standard deviations. Time points with significant change compared to the baseline was shown with asterisks (*p* < 0.05, one-way ANOVA with Dunnett correction).

During the last minute of running, the subjects could perform moderate intensity exercise based on ACSM, 2017: $\dot{V}O_2$ 51.79 ± 4.63%$\dot{V}O_{2peak}$, HR 67.53 ± 4.31%HR_{max}, RPE 10.17 ± 1.94. The SBF was observed significant increase from 9 min after beginning of running until 1min of recovery period. HR and $ETCO_2$ showed similar response in that significant increase

was found from 2 min after beginning of running until 1 min and 3 min of recovery period respectively.

Taken together, all non-cortical physiological variables were observed significant change during running and returned to the baseline within 4 min in recovery period. However, an individual difference in recovery time was found. In slowest recovery case, the subject took 14 min to completely return the physiological variables to the baseline. Therefore, in our point of view, 15 min after finishing running was the appropriate time to start measuring cortical hemodynamic by fNIRS to avoid possible signal contamination (Main text, Fig. 6).

Supplementary D

Short Report for McNemar Test (Modified after Siegel, Sidney & Castellan, 1988)

Origin: first introduced by McNemar, Quinn in 1947

General use of this test: to test the difference between two associated* proportions/frequencies in 2 x 2 dichotomous variables (can be repeated / matched measure) where an ordinary chi-square test is inappropriate due to the violation of assumptions of independence.

*This test can be also used for independent observations.

Advantages of using this method in the currently submitted article:

1. Because a large variability within participants and restricted range-like problem after the treatments due to the nature of the manipulation made the association ambiguous, it was not appropriate and robust to use parametric tests such as Pearson r for the data.
2. In addition, observations of not strong interest (ones with no changes between pre and post treatment) appeared to distort the results for ordinary nonparametric tests such as Spearman rank-order correlation and Kendall's tau.
3. As a result, McNemar test was employed because directionality of the variability in the data was consistent despite the problems discussed above, and McNemar test was able to eliminate influences from uninterested observations (diagonal cells A & D in Table 1 below).

Data format:

Table 1. Data format for McNemar test

		Pre		
		1	0	Total
	1	A	B	A+B
Post	0	C	D	C+D
	total	A+C	B+D	A+B+C+D

Notes: frequency of positive (1) and change (0) infection pre and post the medication

Assumptions for McNemar test:

1. Variables should be dichotomous where diagonal (A & D) represent unchanged observations.
2. It is commonly used for repeated / matched measures*.
   *This test can be also used for independent observations.
3. Sample size: summed frequency at diagonal A + D should be larger than 10.

Hypothesis to be tested:

H_0: p (pre = 0, post = 1) = p (pre = 1, post = 0)

H_1: p (pre = 0, post = 1) $\neq$ p (pre = 1, post = 0)

Formula for $Z_{McNemar}$ and $\chi^2_{McNemar}$:

$$Z_{McNemar} = \frac{|\text{FreqB - FrecC}| - 1}{\sqrt{\text{FreqB + FrecC}}}, \text{ df = (rows - 1) (columns - 1) = 1}$$

$$\chi^2_{McNemar} = \frac{(|\text{FreqB - FrecC}| -1)^2}{\text{FreqB +FreqC}}, \text{ df = (rows - 1) (columns - 1) = 1}$$

Problem and justification to use this method: Since this test compares p(pre = 1, post = 0) and p(pre = 0, post = 1), it ignores the effects of two other probabilities p(pre = 1, post = 1) =and p(pre = 0, post = 0). As shown below, data from Table 5 and Table 6 indicates the same significance level, but interpretation of result would be different. As opposed to the data from Table 4, the data 2's effect is actually larger. In other words, ratio of decreasing positive infection is larger compared with remaining cells. However, in the current study, our purpose was to find the directionality of change after the treatment ignoring the observations that did not change between pre and post treatments, this test fitted our aim.

References

1. American College of Sports Medicine. *ACSM's Guidelines for Exercise Testing and Prescription*. *Medicine & Science in Sports & Exercise* (Lippincott Williams & Wilkins, 2017).
2. Harling, S. A., Tong, R. J. & Mickleborough, T. D. The oxygen uptake response running to exhaustion at peak treadmill speed. *Med. Sci. Sports Exerc.* **35**, 663–668 (2003).
3. Borg, G. Psychophysical bases of perceived exertion. *Med. Sci. Sports Exerc.* **14**, 377–381 (1982).
4. Harms, C. A. *et al.* Effects of respiratory muscle work on cardiac output and its distribution during maximal exercise. *J. Appl. Physiol.* **85**, 609–618 (1998).
5. Hanson, N. J. *et al.* Modality determines VO2max achieved in self-paced exercise tests: validation with the Bruce protocol. *Eur. J. Appl. Physiol.* **116**, 1313–1319 (2016).
6. Morgan DW, Baldini FD, Martin PE, K. W. Ten kilometer performance and predicted velocity at VO2max among well-trained male runners. *Med. Sci. Sport. Exerc.* **21**, 78–83 (1989).
7. Byun, K., Hyodo, K., Suwabe, K., Fukuie, T. & Soya, H. Possible neurophysiological mechanisms for mild-exercise-enhanced executive function: An fNIRS neuroimaging study. *J. Phys. Fit. Sport. Med.* **5**, 361–367 (2016).
8. Shibutani, K. *et al.* Do changes in end-tidal PCO2 quantitatively reflect changes in cardiac output? *Anesth. Analg.* **79**, 829–833 (1994).
